# PPARG as therapeutic target for antifibrotic therapy

**DOI:** 10.17179/excli2020-1136

**Published:** 2020-02-28

**Authors:** Ahmed Ghallab, Abdellatief Seddek

**Affiliations:** 1 Department of Forensic Medicine and Toxicology, Faculty of Veterinary Medicine, South Valley University, Qena 83523, Egypt

## ⁯

***Dear Editor,***

Prevalence and mortality of liver fibrosis continue to grow (Pimpin et al., 2018[17]; Weiskirchen and Tacke, 2016[24]; Leist et al., 2017[13]; Godoy et al., 2013[6]). Liver fibrosis occurs as a consequence of chronic liver damage due to various causes, such as viral infections, super-nutrition, metabolic disorders or genetic diseases (Godoy et al., 2013[6]; Ghallab et al., 2019[5]). 

Recently, Winkler and colleagues performed a comprehensive study to analyze the role of miRNA in liver fibrosis and the development of hepatocellular cancer (Winkler et al., 2020[25]). For this purpose, the authors generated mice that express a constitutively active variant of serum response factor (SRF) in the liver (Winkler et al., 2020[25]). SRF regulates numerous biological processes (Olson and Nordheim 2010[16]; Ohrnberger et al., 2015[15]) and the mice develop hyperproliferative nodules that progress to HCC (Ohrnberger et al., 2015[15]). In this murine HCC model, the authors identified eight miRNA hubs and 54 target genes that regulate components of the fibrotic extracellular matrix (Winkler et al., 2020[25]). Hubs are defined as nodes in a transcriptional regulatory network with an unusual high number of connections (Anastasiadou et al., 2018[2]). Here, the miRNA families let-7, miR-30 as well as miR-29c, miR-335 and miR-338 represent central antifibrotic miRNAs (Winkler et al., 2020[25]). Importantly, these antifibrotic miRNAs (with the exception of miR-335) are regulated by the transcription factor PPARG (Winkler et al., 2020[25]). Therefore, the authors conclude that stimulating this transcription factor may represent a strategy for antifibrotic therapy. 

Currently, numerous research activities are performed to identify or optimize therapies for chronic liver disease (Trauner et al., 2017[22]; Svinka et al., 2017[20]; Ghallab et al., 2016[3]; Schliess et al., 2014[18]). A particular challenge are the different etiologies with toxic (Grinberg et al., 2014[8]; 2018[7]; Albrecht et al., 2019[1]; Sezgin et al., 2018[19]), viral (Kazankov et al., 2014[12]; Theise et al., 2018[21]; Maponga et al., 2018[14]), cholestatic (Vartak et al., 2016[23]; Ghallab et al., 2019[4]; Hessel-Pras et al., 2020[9]) and genetic (Hudert et al., 2019[10]; Jansen et al., 2017[11]) mechanisms. A strength of the present study of Winkler et al.[25] is that the authors have identified hubs to target numerous antifibrotic genes simultaneously, independent of the etiology. Future studies will show, whether hub-targeting therapies will indeed ameliorate fibrosis and delay progression to HCC in mouse tumor models. 

## Conflict of interest

The authors declare no conflict of interest.

## References

[1] Albrecht W, Kappenberg F, Brecklinghaus T, Stoeber R, Marchan R, Zhang M (2019). Prediction of human drug-induced liver injury (DILI) in relation to oral doses and blood concentrations. Arch Toxicol.

[2] Anastasiadou E, Jacob LS, Slack FJ (2018). Non-coding RNA networks in cancer. Nat Rev Cancer.

[3] Ghallab A, Cellière G, Henkel SG, Driesch D, Hoehme S, Hofmann U (2016). Model-guided identification of a therapeutic strategy to reduce hyperammonemia in liver diseases. J Hepatol.

[4] Ghallab A, Hofmann U, Sezgin S, Vartak N, Hassan R, Zaza A (2019). Bile microinfarcts in cholestasis are initiated by rupture of the apical hepatocyte membrane and cause shunting of bile to sinusoidal blood. Hepatology.

[5] Ghallab A, Myllys M, Holland CH, Zaza A, Murad W, Hassan R (2019). Influence of liver fibrosis on lobular zonation. Cells.

[6] Godoy P, Hewitt NJ, Albrecht U, Andersen ME, Ansari N, Bhattacharya S (2013). Recent advances in 2D and 3D in vitro systems using primary hepatocytes, alternative hepatocyte sources and non-parenchymal liver cells and their use in investigating mechanisms of hepatotoxicity, cell signaling and ADME. Arch Toxicol.

[7] Grinberg M, Stöber RM, Albrecht W, Edlund K, Schug M, Godoy P (2018). Toxicogenomics directory of rat hepatotoxicants in vivo and in cultivated hepatocytes. Arch Toxicol.

[8] Grinberg M, Stöber RM, Edlund K, Rempel E, Godoy P, Reif R (2014). Toxicogenomics directory of chemically exposed human hepatocytes. Arch Toxicol.

[9] Hessel-Pras S, Braeuning A, Guenther G, Adawy A, Enge AM, Ebmeyer J (2020). The pyrrolizidine alkaloid senecionine induces CYP-dependent destruction of sinusoidal endothelial cells and cholestasis in mice. Arch Toxicol.

[10] Hudert CA, Selinski S, Rudolph B, Bläker H, Loddenkemper C, Thielhorn R (2019). Genetic determinants of steatosis and fibrosis progression in paediatric non-alcoholic fatty liver disease. Liver Int.

[11] Jansen PL, Ghallab A, Vartak N, Reif R, Schaap FG, Hampe J (2017). The ascending pathophysiology of cholestatic liver disease. Hepatology.

[12] Kazankov K, Barrera F, Møller HJ, Bibby BM, Vilstrup H, George J (2014). Soluble CD163, a macrophage activation marker, is independently associated with fibrosis in patients with chronic viral hepatitis B and C. Hepatology.

[13] Leist M, Ghallab A, Graepel R, Marchan R, Hassan R, Bennekou SH (2017). Adverse outcome pathways: opportunities, limitations and open questions. Arch Toxicol.

[14] Maponga TG, Andersson MI, van Rensburg CJ, Arends JE, Taljaard J, Preiser W (2018). HBV and HIV viral load but not microbial translocation or immune activation are associated with liver fibrosis among patients in South Africa. BMC Infect Dis.

[15] Ohrnberger S, Thavamani A, Braeuning A, Lipka DB, Kirilov M, Geffers R (2015). Dysregulated serum response factor triggers formation of hepatocellular carcinoma. Hepatology.

[16] Olson EN, Nordheim A (2010). Linking actin dynamics and gene transcription to drive cellular motile functions. Nat Rev Mol Cell Biol.

[17] Pimpin L, Cortez-Pinto H, Negro F, Corbould E, Lazarus JV, Webber L (2018). Burden of liver disease in Europe: Epidemiology and analysis of risk factors to identify prevention policies. J Hepatol.

[18] Schliess F, Hoehme S, Henkel SG, Ghallab A, Driesch D, Böttger J (2014). Integrated metabolic spatial-temporal model for the prediction of ammonia detoxification during liver damage and regeneration. Hepatology.

[19] Sezgin S, Hassan R, Zühlke S, Kuepfer L, Hengstler JG, Spiteller M (2018). Spatio-temporal visualization of the distribution of acetaminophen as well as its metabolites and adducts in mouse livers by MALDI MSI. Arch Toxicol.

[20] Svinka J, Pflügler S, Mair M, Marschall HU, Hengstler JG, Stiedl P (2017). Epidermal growth factor signaling protects from cholestatic liver injury and fibrosis. J Mol Med (Berl).

[21] Theise ND, Jia J, Sun Y, Wee A, You H (2018). Progression and regression of fibrosis in viral hepatitis in the treatment era: the Beijing classification. Mod Pathol.

[22] Trauner M, Fuchs CD, Halilbasic E, Paumgartner G (2017). New therapeutic concepts in bile acid transport and signaling for management of cholestasis. Hepatology.

[23] Vartak N, Damle-Vartak A, Richter B, Dirsch O, Dahmen U, Hammad S (2016). Cholestasis-induced adaptive remodeling of interlobular bile ducts. Hepatology.

[24] Weiskirchen R, Tacke F (2016). Liver fibrosis: Which mechanisms matter?. Clin Liver Dis (Hoboken).

[25] Winkler I, Bitter C, Winkler S, Weichenhan D, Thavamani A, Hengstler JG (2020). Identification of Pparγ-modulated miRNA hubs that target the fibrotic tumor microenvironment. Proc Natl Acad Sci U S A.

